# Supplementary material for: WordCommentsAnalyzer: A windows software tool for qualitative research
Source: F1000Res. 2018 Sep 4;7:536. Originally published 2018 May 3. [Version 2] doi: 10.12688/f1000research.14819.2 (PMC6137411; doi:10.12688/f1000research.14819.2)
Supplement: Supplementary file 3 [file f1000research-7-17614-s0002.tgz › b2a79496-4ada-47c4-9682-a99ce867836d.zip!/Eight Iranian Health Journals/Journal of Research in Rehabilitation Sciences _28.docx]

© Journal of Research in Rehabilitation Sciences . This work is licensed under CC BY-NC 4.0 (https://creativecommons.org/licenses/by-nc/4.0/).

Volume & Issue: دوره 9، شماره 5: 1392

Title: مقایسه پاسخ های میان رس شنوایی در افراد دو زبانه ترکی-فارسی با افراد تک زبانه فارسی زبان

http://jrrs.mui.ac.ir/index.php/jrrs/article/view/1145

مقدمه : افراد دوزبانه مجبور به كنترل تداخل دوزبان‌ هستند تا از استفاده‌ي غيرعمدي از زبان غير هدف جلوگیری کنند. در سطح زیرقشری، هسته‌های قاعده‌ای و تالاموس در کنترل دو زبان در دوزبانه نقش دارند. از آن‌جا که مولدهای پاسخ‌های میان‌رس شنوایی ((AMLRs نیز در مسیرهای تالاموسی- قشری قرار دارند. هدف این مطالعه مقایسه پاسخ‌های میان‌رس شنوایی افراد دو زبانه زودهنگام آذری _ فارسی با افراد تک زبانه فارسی می‌باشد.    مواد و روش‌ها :این مطالعه مقطعی مقایسه‌ای روی 37 فرد دوزبانه زودهنگام آذری _ فارسی با میانگین سنی77/21 سال و 37 فرد تک زبانه فارسی با میانگین سنی02/22 سال در دو جنس انجام شد. آزمون  AMLRsدر دو گروه با محرک کلیک ثبت شد و دامنه و زمان نهفتگی امواج در دو گروه مورد مقایسه قرار گرفت. همچنین میزان همبستگی استفاده از زبان آذری با اجزا پاسخ‌های میان‌رس شنوایی در افراد دو زبانه بررسی شد.   یافته‌ها: میانگین دامنه Na-Pa با محرک کلیک در گوش راست گروه دوزبانه بیشتر از گروه تک زبانه می‌باشد (04/0=P). میانگین دامنه Pa-Nb درگوش راست (01/0=P) و چپ (03/0=P) گروه دو زبانه بیشتر از گروه تک‌زبانه بود. همچنین بین میزان استفاده از زبان آذری و دامنه Na-Pa (01/0=P) (427/0=r) و  Pa-Nb(04/0=P) (348/0=r) در افراد دو زبانه همبستگی مشاهده شد. نتیجه‌گیری: پتانسیل‏هاي برانگيخته ميان‏رس شنوايي در بزرگسالان از ثبات مطلوبي برخوردار است .از آنجائي که این مطالعه بهبودی را در دامنه AMLRs در افراد دو زبانه که سطح مهارت بالایی در هر دو زبان دارند و به طور متداول در زندگی روزمره از هر دو زبان استفاده می‌کنند را نسبت به تک زبانه‌ها نشان داد، در نظر گرفتن این مورد در آزمون پاسخ‌های میان‌رس شنوایی توصیه می‌شود.

Title: بررسی مصرف انرژی حین راه رفتن در بیماران مبتلا به کمر درد مزمن غیر اختصاصی در مقایسه با افراد سالم بر اساس شاخص مصرف فیزیولوژیکال

http://jrrs.mui.ac.ir/index.php/jrrs/article/view/1221

مقدمه: کمردرد وضعیت شایع و پیچیده ای است که در برنامه‌ریزی‌های درمانی با دشواری‌هایی همراه است که باعث فشارهای اقتصادی بزرگی روی افراد، خانواده‌ها، جوامع و دولت‌ها می‌شود. راه رفتن یکی از اصلی‌ترین و کارآمدترین فعالیت‌های روزانه انسان است که برای داشتن یک زندگی مستقل ضروری است. حال به نظر می‌رسد افرادی که دچار اختلالات عضلانی ـ اسکلتی هستند، مانند مبتلایان به کمردرد مزمن غیراختصاصی، نسبت به افراد سالم انرژی بیشتری صرف راه رفتن می‌کنند. بنابراین، هدف این مطالعه تعیین مصرف انرژی مبتلایان به کمردرد مزمن غیر اختصاصی در مقایسه با افراد سالم حین راه رفتن است. مواد و روش‌ها: مطالعه حاضر از نوع مقطعی ـ مقایسه‌ای می‌باشد. مطالعه برروی دو گروه 16 نفره سالم و بیمار در بهار 92 انجام گرفت. در این مطالعه از شاخص مصرف فیزیولوژیکال به عنوان یک ابزار کلینیکی برای سنجش مصرف انرژی حین فعالیت، به ویژه راه رفتن، استفاده شد. تفاوت بین مقادیر میانگین سرعت راه رفتن و شاخص مصرف فیزیولوژیکال در دوگروه، با استفاده از آزمون T محاسبه گردید. یافته‌ها: تفاوت ضربان قلب در افراد سالم و بیمار، حین استراحت و راه رفتن معنی‌دار بود. میانگین سرعت راه رفتن در افراد سالم 7/5± 05/76 متر در دقیقه در مقایسه با 83/7±17/69 متر در دقیقه در افراد بیمار بود (006/0P=). همچنین، شاخص مصرف فیزیولوژیکال بین دو گروه بررسی شده از تفاوت معنی‌داری برخوردار بود که در گروه سالم 06/0±289/0 ضربه در متر و در گروه بیمار 098/0±388/0 ضربه در متر به دست آمد. نتیجه‌گیری: نتایج مطالعه نشان می‌دهد که مصرف انرژی این بیماران به دلیل کاهش سرعت راه رفتن یا ناکارآمدی عضله قلب، که خود ناشی از کم تحرکی است، بیشتر از افراد عادی است.

Title: پایایی آزمون– بازآزمون نسخه فارسی شده «شاخص استقلال در فعالیت های روزمره زندگی کَتز» در افراد سکته مغزی حاد ایرانی

http://jrrs.mui.ac.ir/index.php/jrrs/article/view/1058

مقدمه: سکته مغزی یکی از عمده‌ترین بیماری‌هایی است که منجر به افزایش شمار افراد مبتلا به نقایص حسی- حرکتی و فقدان عملکرد می‌گردد که به دنبال آن درمان‌های توانبخشی برای بهبود بیمار ضرورت پیدا می‌کند. از مهم‌ترین اهداف توانبخشی، ارزیابی عملکرد روزمره زندگی فرد می‌باشد.هدف این مطالعه بررسی پایایی آزمون– بازآزمون نسخه فارسی مقیاس فعالیت‌های روزمره زندگی کَتزدر افراد سکته مغزی حاد ایرانی می‌باشد. مواد و روش‌ها: نسخه فارسی شاخص کَتز روی 60 مراجع سکته مغزی حاد، توسط کارشناس کاردرمانی اجرا شد. ارزیابی آزمون­گر طی دو مرتبه و به فاصله چهارده روز(دو هفته) صورت گرفت. پایایی توسط همخوانی درونی(ضریب آلفای کرونباخ) و آزمون– بازآزمون(ضریب همبستگیICC, MDC, SEMو Kappa) محاسبه و تعیین شد. یافته‌ها: همخوانی مقیاس ثابت کرد که ضریب آلفای کرونباخ (787/0) در مرحله اول و (783/0) در مرحله دوم ارزیابی شاخص کَتز قابل قبول می‌باشد. ضریب همبستگی ICC در آزمون- بازآزمون برای نمره کل شاخص کَتز 811/0 سنجیده شد که از پایایی بسیار مطلوب برخوردار است ولی  SEMبا 68%  اطمینان بیشتر از 10% بالاترین نمره ممکن برای شاخص کَتز بود (مقادیر کمتر از 10% قابل قبول می‌باشد) و همچنین در بررسی میزان توافق آزمون- بازآزمون در اکثر آیتم‌ها، سطح توافق متوسط تا خوبی مشاهده شد.MDC نشان داد که به ازای 337/2 کمترین تغییر در شاخص کَتز به دست می‌آید. نتیجه‌گیری: نتایج پیشنهاد می‌کند که نسخه فارسی شاخص کَتز اجرا شده برای افراد سکته مغزی حاد ایرانی یک وسیله ارزیابی پایا می‌باشد.

Title: تعيين پايايي و روايي ترجمه فارسي پرسشنامه آنژين سياتل (SAQ) در بيماران سکته قلبي

http://jrrs.mui.ac.ir/index.php/jrrs/article/view/1234

مقدمه : سکته قلبي (Myocardial Infarction: MI) علت اصلي مرگ و مير در دنياي صنعتي مي‌باشد. سکته قلبي علاوه تحميل هزينه‌هاي اقتصادي و درماني، روي کيفيت زندگي مرتبط با سلامت هم اثر مي‌گذارد. نسخه اصلي پرسشنامه SAQ به صورت مقطعي ـ زماني تهيه شده و به صورت کلينيکي براي اندازه‌گيري ابعاد مهم بيماري عروق کرونري استفاده مي‌شود ولي ترجمه فارسي آن موجود نيست. مواد و روش‌ها: پس از ترجمه چند مرحله‌اي مطابق با پروتکل Manufacturers Alliance for Productivity and Innovation (MAPI)، پايايي ابزار روي 130 فرد مبتلا به MI مورد بررسي گرفت. به منظور بررسي روايي ابزار از يک پرسشنامه فرم کوتاه 36 (Short Form-36: SF-36) استفاده شد. بيماران جهت تعيين تكرارپذيري، پرسشنامه SAQ را پس از 5 روز مجدداً پر كردند. یافته‌ها: پايايي در اين مطالعه با استفاده از روش‌هاي آماري Intra Class Correlation (ICC) و آلفاي کرونباخ مورد بررسي قرار گرفت. روايي ابزار با استفاده از ضريب همبستگي پيرسون براي نمره کل آزمون و سؤالات تعيين شد. در تمام ابعاد پرسشنامه ICC بيشتر از 6/0 به دست آمد. کاپاي وزني بين 492/0 تا 853/0 بود. نتیجه‌گیری: پايايي و روايي نسخه فارسي سياتل آنژين در اين مطالعه قابل قبول است و به نظر مي‌رسد اين پرسشنامه مي‌تواند ابزار مناسبي در بررسي کيفيت زندگي مرتبط با سلامت در بيماران فارسي زبان سکته قلبي با شکايت آنژين باشد

Title: تجارب دانشجویان فیزیوتراپی از فرایندهای آموزش بالینی: مطالعه کیفی

http://jrrs.mui.ac.ir/index.php/jrrs/article/view/1092

مقدمه: امروزه جایگاه آموزش بالینی در تربیت دانشجویان فیزیوتراپی بطور فزاینده‌ای در حال برجسته‌تر شدن است. برنامه‌ریزی صحیح در تربیت بالینی دانشجویان مستلزم کسب شناختی درست از تجارب دانشجویان می‌باشد. این پژوهش با هدف کسب شناختی بهتر از  تجارب دانشجویان فیزیوتراپی از فرایندهای جاری در دورة آموزش بالینی انجام پذیرفته است. مواد و روش‌ها: در این مطالعه کیفی، 21 نفر از دانشجویان سال آخر مقطع کارشناسی فیزیوتراپی بصورت نمونه‌گیری هدفمند به مشارکت در پژوهش دعوت و اطلاعات از طریق پنج جلسه بحث گروهی متمرکز جمع‌آوری شد. از روش کلایزی به عنوان راهنمای تجزیه و تحلیل استفاده و روایی یافته‌ها به روش مثلث‌سازی داده‌ها تأمین گردیده است. یافته‌ها: پس از تجزیه و تحلیل داده‌ها و طبقه‌بندی کدها، چهار مضمون شامل «آموزش و ارزشیابی بالینی»، «یادگیری بالینی»، «پذیرش بیمار» و «ارتباطات کارآموز در محیط» پدیدار گشت. مشارکت‌کنندگان طی این مطالعه بر مواردی مانند تعداد و تنوع کم بیماران، عدم اجرای سرفصل‌های کارآموزی و جمعیت زیاد گروه‌های کارآموزی تأکید داشتند. نتیجه‌گیری: بی‌توجهی به فرایندهای جاری در آموزش بالینی، تجربة بالینی کارآموز را متأثر می‌سازد لذا بهبود فرایندهای کارآموزی، تجربة بالینی موثرتری را فراهم می‌آورد. بالابردن غنای تجربة بالینی کارآموزان با تأمین تنوع کافی بیمار در کارآموزی‌ها، افزایش دورة آموزش عملی و بالینی، تأکید بیشتر و عملی بر اجرای بهتر سرفصل‌های کارآموزی، تعدیل جمعیت دانشجویان در کارآموزی برای اثربخش‌تر شدن کارآموزی‌ها پیشنهاد می‌گردد.

Title: بررسی مقایسه ای نتایج آزمون همخوان- واکه دایکوتیک در بزرگسالان راست دست و چپ دست دارای شنوایی هنجار

http://jrrs.mui.ac.ir/index.php/jrrs/article/view/1319

مقدمه: بر طبق پژوهش‌های انجام شده، نیمکره چپ مغز برای پردازش زبان اختصاصی شده است. رابطه بین برتری دستی و نیمکرات مغزی بسیار بدیهی بوده و بر کسی پوشیده نیست، به طور کلی در بیش از 95%  افراد راست دست و 70% افراد چپ دست نیمکره چپ مغز مسؤول پردازش اطلاعات زبانی است. مطالعات اندکی به بررسی رابطه بین برتری دستی با نیمکره‌های مغزی با استفاده از آزمون همخوان واکه دایکوتیک ( DCV ) پرداخته‌اند. هدف از این مطالعه، بررسی مقایسه‌ای نتایج آزمون همخوان- واکه دایکوتیک در بزرگسالان راست دست و چپ دست دارای شنوایی هنجار می‌باشد. مواد و روش‌ها: در این مطالعه، 40 فرد بزرگسال راست دست و 40 فرد بزرگسال چپ دست در سنین 40-18 سال که با استفاده از مقیاس برتری دستی ادینبرگ راست دستی یا چپ دستی آن‌ها تائید شده بود شرکت کردند. پس از انجام آزمون DCV در 3 مرحله،در محیط نرم‌افزار SPSS  نسخه 18 برای تجزیه و تحلیل داده‌های نرمال که شامل امتیاز فرد در حالت عدم همزمان ارائه محرک در دو گوش است از آزمون t- testزوجی و برای تجزیه و تحلیل داده‌های غیر نرمال از قبیل امتیاز گوش راست و چپ در حالت ارایه همزمان محرک در دو گوش و تعداد جفت همخوان- واکه‌های درست تشخیص داده شده در دو گوش از آزمون Mann- Whitney استفاده گردید. یافته‌ها: بر اساس نتایج به دست آمده در این مطالعه، امتیاز کسب شده در هر 3 مرحله توسط گروه راست دست بیشتر از امتیاز کسب شده توسط گروه چپ دست بود که از لحاظ آماری معنی‌دار می‌باشد ( P≤ 0.01 ). درصد تعداد جفت همخوان واکه‌های درست تشخیص داده شده در گروه راست دست نیز بیشتر از گروه چپ دست بود. نتیجه‌گیری: نتایج آزمون همخوان واکه دایکوتیک ( DCV ) دال بر عملکرد بهتر افراد راست دست نسبت به افراد چپ دست می‌باشد. برتری گوش راست نه تنها در گروه راست دست بلکه در گروه چپ دست نیز مشهود است این مسأله برتری نیمکره چپ مغزی را در پردازش محرک‌های گفتاری نشان می‌دهد.

Title: اثرات کاپينگ درمانی بر روی خواص بيومکانيکی بافت‌های جلدی در موش صحرايی

http://jrrs.mui.ac.ir/index.php/jrrs/article/view/1193

مقدمه: کاپينگ درمانی به عنوان يکی از روش‌های درمانی رايج به صورت گسترده ای در اکثر کشورهای آسيايی جهت درمان بالينی ضايعات بافت نرم مورد استفاده قرار گرفته است، ولی شواهد پايه ای قوی در تاٌييد آن وجود ندارد. يکی از اثرات احتمالي کاپينگ اثر بر روی ويژگيهای بيومکانيکی بافت‌های تحت كاپينگ می‌باشد. هدف از اين مطالعه، بررسی بنيادی اثرات کاپينگ درمانی بر روي برخی ويژگيهای بيومکانيکی پوست شامل سفتی و حداکثر قدرت کششی در موش صحرايی می‌باشد. مواد و روش‌ها: در اين مطالعه تجربی، در مجموع 20 موش صحرايی (10 نمونه جهت کاپينگ درمانی و 10 نمونه به عنوان گروه کنترل خالص) به روش نمونه گيری غير تصادفی ساده در دسترس از حيوان خانه بخش آناتومی دانشگاه علوم پزشکی تهران انتخاب شدند. سفتی و حداکثر قدرت کششی پوست تحت کاپينگ به مدت 10 دقيقه در طی 12 روز متوالی در اين نمونه‌ها در 3 گروه آزمون، کنترل آزمون و کنترل خالص توسط دستگاه تنسيوتر مورد ارزيابی قرار گرفت. جهت تحليل داده‌ها از آزمون تی زوجی برای مقايسه پوست طرفين هر نمونه در گروه آزمون و از آزمون تی مستقل برای مقايسه بين گروه‌ها استفاده گرديد.  یافته‌ها: ميزان سفتی و حداکثر قدرت کششی پوست گروه آزمون (کاپينگ) نسبت به پوست گروه کنترل آزمون و گروه کنترل خالص به طور معنی داری کاهش يافت (به ترتيب 048/0P=، 029/0P=، 018/0P= و 008/0P=). نتیجه‌گیری: کاپينگ درمانی می‌تواند در ايجاد تغييراتی در ويژگيهای بيومکانيکی پوست موش صحرايی شامل کاهش سفتی و حداکثر قدرت کششی موٌثر ‌باشد.

Title: مقایسه سلامت روانی در مردان سالمند دارای سطوح متفاوت فعالیت ورزشی هفتگی

http://jrrs.mui.ac.ir/index.php/jrrs/article/view/993

مقدمه:اولينگامجهتپيشگيريازبيماري‌هايمزمنوبهبود سطح سلامتی سالمندان، شناسايي وضعیت سلامتی در آنها است. هدف پژوهش حاضر مقایسه سلامت روانی در مردان سالمند دارای سطوح متفاوت فعالیت ورزشی هفتگی بود. مواد و روش‎ها:آزمودنی‌های تحقیق حاضر را تعداد 300 نفر از مردان سالمند بالای 65 سال شهر تهران که دارای سطوح متفاوتی از فعالیت ورزشی هفتگی (بالا، متوسط و پایین) بودند، تشکیل داد. ابزار جمع‌آوری اطلاعات در مطالعه حاضر پرسشنامه سلامت روانی گلدبرگ (GMH-28) بود و پایایی آن نیز از طریق ضریب آلفای کرونباخ (86/0 =α) کسب شد. روش‌های آماری شامل آزمون K-S به منظور تعیین نرمال بودن داده‌ها، آزمون ANOVA جهت مقایسه سلامت روانی در مردان سالمند دارای سطوح متفاوت فعالیت‌های جسمانی هفتگی و در نهایت آزمون تعقیبی توکی جهت بررسی تفاوت میانگین گروه‌های مورد بررسی استفاده شد. یافته‌ها:نتایج مربوط به آزمون ANONA نشان داد که بین سلامت روانی مردان سالمند دارای سطوح متفاوت فعالیت ورزشی هفتگیتفاوت معنی‌داری وجود دارد (001/0P=). علاوه بر این، نتایج مربوط به آزمون تعقیبی توکی نشان داد که بین سلامت روانی مردان سالمند دارای سطوح بالای فعالیت هفتگی با مردان سالمند دارای سطوح پایین(00/0P=) و همچنین مردان سالمند دارای سطوح متوسط فعالیت هفتگی با مردان سالمند دارای سطح پایین(001/0P=)تفاوت معنی‌داری وجود دارد. یافته‎ جالب توجه این تحقیق عدم مشاهده‎ تفاوت معنی‌دار (59/0P=) بین سلامت روانی مردان سالمند دارای سطوح بالا و متوسط فعالیت هفتگی بود. نتيجه‌گيری:با توجه به اثربخشی تمرین جسمانی برای سلامت روانی افراد سالمند و ارتباط فعالیت‌های ورزشی متوسط هفتگی با سلامت روانی آن‎ها، پیشنهاد می‌شود که برنامه‌های خاص ورزشی به منظور ارتقای سلامتی مردان سالمند اتخاذ گردد.

Title: ارزیابی عوامل مؤثر در پوشش پذيري وزوز

http://jrrs.mui.ac.ir/index.php/jrrs/article/view/1009

مقدمه: وزوز يكي از نشانه‌هاي شايع بيماران مراجعه‌كننده به كلینيك‌هاي شنوايي‌شناسي مي‌باشد كه بيش از 10 درصد جمعيت بزرگسال به آن مبتلا هستند. يكي از ويژگي‌هاي مهم وزوز قابليت پوشش‌پذيري آن است كه به عنوان يك روش درمان فيزيولوژيك تلقي مي‌شود. در اين مطالعه نقش عوامل مختلف مثل بلندي و فركانس وزوز، ميزان كم‌شنوايي و محل احساس وزوز در قابليت پوشش‌پذيري مورد بررسي قرار گرفته است. مواد و روش‌ها: اين مطالعه توصيفي- تحليلي به صورت مقطعي در 45 بيمار كه با شكايت وزوز به كلنيك شنوايي‌شناسي دانشكده علوم توانبخشي مراجعه داشتند، انجام شده است. در اين افراد آزمايشات اديومتري ارزيابي وزوز شامل تعيين بلندي و فركانس وزوز، سطوح حداقل پوشش و وقفه پايدار انجام شد. قابليت پوشش‌پذيري براساس نتايج تست وقفه پايدار  و با ارايه محرك نويز باند باريك در شدت 10 dB بالاتر از سطوح حداقل پوشش تعيين گرديد. كليه آزمايشات با دستگاه اديومتر Madsen مدل OB822 انجام شد. یافته‌ها: بر اساس نتايج در 7/46 درصد افراد مورد بررسي وزوز داراي پوشش‌پذيري كامل و 6/35 درصد افراد پوشش‌پذيري جزئي داشتند و تنها در 6/17 درصد افراد وزوز غير قابل پوشش بود. همچنين يافته‌ها نشان داد كه بين بلندي وزوز و قابليت پوشش‌پذيري آن رابطه وجود دارد. با توجه به دیگر نتایج بين قابليت پوشش‌پذيري، فركانس وزوز و ميزان كم‌شنوايي ارتباط معنی‌داری مشاهده نشد. يافته‌ها همچنین نشان دادند كه بين محل احساس وزوز و قابليت پوشش‌پذيري آن ارتباطي وجود ندارد. نتیجه‌گیری: با توجه به نتايج اين مطالعه وزوز در درصد قابل توجهي از افراد پوشش‌پذير است و از اين شيوه مي‌توان جهت كنترل و تخفيف علايم استفاده نمود.

Title: مقایسه برخی مهارت های نحوی افراد مبتلا به مالتیپل اسکلروزیس عود کننده بهبود یابنده و افراد طبیعی فارسی زبان استان اصفهان

http://jrrs.mui.ac.ir/index.php/jrrs/article/view/1220

مقدمه: مالتیپل اسکلروزیس بیماری پیشرونده سیستم عصبی مرکزی است که غلاف میلین را تخریب می‌کند و پلاک در ماده سفید مغز و نخاع تولید می‌کند . مطالعات نشان داده‌اند مهارت‌های زبانی در این بیماری تحت تاثیرقرار می‌گیرد. آسیب‌شناسان گفتار و زبان توجه اندکی به مهارت­های زبانی این بیماران معطوف داشته‌اند. هدف مطالعه حاضر مقایسه برخی از مهارت‌های نحوی بزرگسالان مبتلا به مالتیپل اسکلروزیس عود کننده- بهبودیابنده و بزرگسالان طبیعی فارسی زبان استان اصفهان بود . مواد و روش‌ها: در این مطالعه توصیفی-تحلیلی و مقطعی 17 بزرگسال مبتلا به مالتیپل اسکلروزیس عود کننده- بهبودیابنده از مرکز MS بیمارستان آیت‌ا... کاشانی، وابسته به دانشگاه علوم پزشکی اصفهان و 34 بزرگسال طبیعی که از نظر سن، جنس، تحصیلات با گروه بیمار مطابق بودند مورد بررسی قرار گرفتند. از افراد درصورت داشتن معیار ورود، نمونه گفتاري درمورد تاريخچه زندگي گرفته شد. اين نمونه گفتار ضبط و نوشته شد. سپس داده‌ها تعیین و مقایسه انجام گردید. یافته‌ها: در این مطالعه بین میانگین طولانی‌ترین طول جمله (043/ P value =) و تعداد کل جمله ( 001/ P value =) افراد سالم و بیمار تفاوت معنی‌داری یافت شد. در متغیرهای میانگین طول جمله و نسبت کاربرد جملات ساده و نسبت کاربرد جملات مرکب تفاوت معنی‌داری بین دو گروه وجود نداشت. اگرچه بیماران حاضر در این مطالعه به درجات خفیفی از بیماری (میانگین EDSS =35/1) مبتلا بودند اما تفاوت‌هایی در بعضی متغیرهای نحوی با افراد سالم دارند. نتیجه‌گیری: این یافته بر نیاز به ارزیابی زبان  بیماران MS تأکید می‌کند. با توجه به محدودیت‌هایی که آسیب‌شناسان گفتار و زبان با توجه به نداشتن آزمون استاندارد برای ارزیابی این بیماران دارند این پژوهش نشان داد که گرفتن تک‌گویی روشی مفید برای ارزیابی این بیماران است.

Title: بررسی اثر خستگی عضلات کمر بر تغییرات ضخامت عضلات شکمی به دنبال تكليف باربرداری در زنان سالم

http://jrrs.mui.ac.ir/index.php/jrrs/article/view/1163

مقدمه: خستگی عضلات کمر یکی از عوامل خطرزا در محیط کار و زندگی محسوب می‌شود. در شرایط خستگی، بار بین بافت‌های فعال و غیرفعال به صورت غیربهینه پخش می‌شود که ممکن است خطر آسیب حتی با اعمال بار کم را نیز به دنبال داشته باشد. با توجه به اهمیت عضلات شکمی در ایجاد و حفظ پایداری ستون فقرات در شرایط ناپایدار بررسی این عضلات در مقابله با خستگی عضلات کمر ضروری به نظر می‌رسد. با توجه به اینکه بررسی عضلات عمقی با روش الکترومیوگرافی سطحی امکان‌پذیر نیست و در این مطالعه روش تصویربرداری اولتراسونیک روش جایگزین مناسبی برای بررسی این عضلات در نظر گرفته شده است. مواد و روش‌ها:پژوهش حاضر یک مطالعه تجربیاست که بر روی 15 زن سالم غیر ورزشکار انجام شده است. ضخامت عضلات شکمی این افراد توسط دستگاه اولتراسوند real-time از بخش لترال شکم ( عضله عرضی شکمTransverseabdominis (TrA)، مایل داخلی شکم Internal oblique abdominis (IO)، مایل خارجی شکم External oblique abdominis (EO)) قبل و بعد از پروتکل خستگی عضلات اکستانسور کمری در حالت ایستاده حین تحمل بار 25% وزن بدن و بدون تحمل این مقدار بار مورد ارزیابی قرار گرفت. آزمون آماریتحلیل واریانس با اندازه‌گیری‌های مکرربرای بررسی اثر دو متغیر بار محوری و خستگی عضلات اکستانسور کمر بر تغییرات ضخامت عضلات شکمی استفاده شد. یافته‌ها:اگر چه بار قرینه محوری به اندازه 25% وزن بدن هر فرد شرکت‌کننده، نتوانست تغییر ضخامت معنی‌داری در هیچ یک از عضلات شکمی ایجاد کند. اما به دنبال  خستگی عضلات اکستانسور کمر افزایش معنی‌دار در ضخامت عضله مایل داخلی شکم ديده شد (009/0P= ). نتیجه‌گیری: نتایج نشان داد به دنبال خستگی عضلات اکستانسور کمر، رفتار عضلات شکمی به صورت افزايش ضخامت عضلات شکمی تغيير مي‌كند تا احتمالا پایداری ستون فقرات در آن وضعيت حفظ شود.

Title: تأثیر فوری استفاده کوتاه مدت از اسپری‌های سردکننده، بر حس وضعیت مفصل مچ پای افراد سالم

http://jrrs.mui.ac.ir/index.php/jrrs/article/view/1228

مقدمه: تاکنون تحقيقات متعددي درخصوص تأثیر سرما بر عملکرد حس وضعيت مفصل مچ پا صورت گرفته است اما پژوهشی که از اسپری‌های سردکننده برای این منظور استفاده کرده باشد یافت نشد. هدف این تحقیق، بررسی اثر فوری یک پروتکل کوتاه مدت اعمال سرمای موضعی بااستفاده از اسپری‌های سردکننده بر حس وضعیت مفصل مچ پای افراد سالم بود. مواد و روش‌ها: 30 فوتبالیست مرد سالم با ميانگين سنی 29/1±2/23 سال، قد 92/5±11/177 سانتي‌متر و وزن 73/6±5/69 كيلوگرم در تحقیق شرکت کردند. اعمال سرما با استفاده از اسپری‌های سردکننده و به طور همزمان از جانب داخل و خارج مچ پا انجام شد. دقت عملکرد حس وضعیت مچ پا، قبل و بلافاصله پس از سرمادهی موضعی واز طریق اندازه‌گیری خطای بازسازی زوایای 10 و 20 درجه دورسی و پلانتار فلکشن توسط الکتروگونیامتر ارزیابی شد. یافته ها: نتایج نشان داد که علی‌رغم کاهش دقت عملکرد حس عمقی مفصل مچ پا پس از اعمال سرمای موضعی به صورت کوتاه مدت، از نظر آماری اختلاف معنی‌داری میان خطای بازسازی زاویه هدف، قبل و بعد از اعمال سرما وجود ندارد. نتیجه‌گیری: به نظر می‌رسدکه استفاده کوتاه مدت از اسپری سردکننده تنها بر گیرنده‌های پوستی تأثیر فوری داشته  دوک‌های عضلانی و گیرنده‌های مفصلی (گیرنده‌های عمقی‌تر) که نقش اصلی در حس وضعیت مفصل دارند تحت تأثیر قرار نگرفته اند.

Title: تهیه و بررسی روایی و پایایی آزمون تکرار ناکلمه و مقایسه حافظه فعال واجی در کودکان عادی و دارای اختلال صداهای گفتار 4 تا 6 ساله ی شهر تهران

http://jrrs.mui.ac.ir/index.php/jrrs/article/view/1161

مقدمه: اختلال صداهای گفتار (Speech Sound Disorders یا SSD) از اختلالات شایع در کودکان است که ویژگی آن خطاهای نامتناسب رشدی در تولید گفتار است که می‌تواند موجب کاهش وضوح گفتار شود. برخی داده‌ها نشان می‌دهد نقص در حافظه فعال واجی ممکن است در این اختلال سهم داشته باشد. حافظه فعال واجی به عنوان بخشی از دستگاه حافظه فعال، مسئول رمزگذاری و ذخیره اطلاعات واجی است. بسیاری از پژوهشگران تکرار ناکلمه (Non-Word Repetition یا NWR) را برای سنجش حافظه فعال واجی به کار گرفته‌اند. هدف این پژوهش تهیه و بررسی روایی و پایایی آزمون تکرار ناکلمه و کشف تفاوت‌های حافظه فعال واجی در کودکان عادی و SSD 4 تا 6 ساله فارسی زبان بوده است. مواد و روش‌ها: شرکت‌کنندگان در این مطالعه 32 کودک SSD و 32 کودک طبیعی- همسان به لحاظ سن و جنس- به عنوان گروه کنترل بودند. حافظه فعال واجی در هر دو گروه با تکلیف تکرار ناکلمه مورد بررسی قرار گرفت. بدین منظور تکلیف NWR طراحی شد و پس از تعیین روایی و پایایی، در هر دو گروه مورد استفاده قرار گرفت. جهت تعیین وجود تفاوت بین میانگین امتیازهای NWR در دو گروه آزمودنی، آزمون Mann-Whitney U مورد استفاده قرار گرفت. یافته‌ها: 25 ناکلمه این آزمون دارای روایی محتوایی هستند. بین اجراهای متوالی ضریب همبستگی 997/0 به دست آمد        (00/0 >P). میانگین امتیاز کودکان SSD در مقایسه با گروه کنترل، پایین‌تر بود و نتایج آزمون آماری نشان داد که میان توانایی تکرار ناکلمه کودکان عادی و SSD تفاوت معنی‌داری وجود دارد (00/0 > P). نتیجه‌گیری: آزمون تکرار ناکلمه از روایی و پایایی بالایی برخوردار بود. عملکرد ضعیف‌تر کودکان SSD در تکرار ناکلمه ممکن است به دلیل ضعف در نگهداری توالی‌های جدید صدا در حافظه فعال واجی باشد که احتمالاً اجازه شکل‌گیری بازنمایی‌های واجی بلند مدت با ثبات را نمی‌دهد. در غیاب عوامل علت‌شناختی آشکار، نقص در حافظه فعال واجی می‌تواند به عنوان علت این اختلال مطرح شود.

Title: مروری بر توانایی عملکردی افراد مبتلا به قطع عضو بالای زانو در راه رفتن با مفصل دارای ریزپردازندهC-Leg و سایر مفاصل پروتزی زانو

http://jrrs.mui.ac.ir/index.php/jrrs/article/view/882

مقدمه:  C-Leg (ساخت شرکت اتوبوک، کشور آلمان) یک مفصل پروتزی زانو میباشد که توسط ریز پردازنده ها کنترل شده و ممکن است منجر به بهبود راه رفتن در افراد مبتلا به قطع عضو شود. مطالعاتی که در مورد کارایی مفاصل پروتزی کنترل شونده با ریز پردازنده ها صورت گرفته است، مجموعه ای از نتایج مبهم را در اختیار خواننده قرار می دهد که از جمله می توان به موارد زیر اشاره کرد: استفاده از مفاصل کنترل شونده توسط ریز پردازنده ها در حین راه رفتن روی سطح صاف منجر به افزایش تقارن راه رفتن، کاهش فشار روی اندام پروتزی و اندام سالم  و همچنین کاهش مصرف انرژی در فرد مبتلا به قطع عضو می شود ، در حالیکه بعضی مطالعات نشان می دهند که هیچ گونه تفاوت خاصی بین مفاصل سنتی و مدرن زانو وجود ندارد.به همین علت تناقضات زیادی در مورد این مسئله که آیا C-Leg  بر عملکرد و توانایی  فرد مبتلا به قطع عضو  تاثیرگذار است یا نه ؟ وجود دارد. هدف تحقیق حاضر بررسی و مرور مطالعات صورت گرفته در زمینه ی ارزیابی  میزان ثبات ، مقدار مصرف انرژی و شاخصه های راه رفتن  در مبتلایان به قطع عضو بالای زانو  که از زانوی پروتزی C-Leg استفاده می کنند، می باشد. مواد و روش‌ها: مقالات مرتبط  با موضوع تحقیق حاضر از طریق جستجو در پایگاههای Medline, ISI web of knowledge and PubMed  و در محدوده زمانی سالهای 1999 الی 2012 شناسایی شد. پس از بررسی و طبقه بندی مطالب بدست آمده ، مقالات موجود برای ارزیابی کیفیت روش مورد استفاده  بر اساس سه پارامتر مهم یعنی میزان ثبات ، مقدار مصرف انرژی و شاخصه های راه رفتن مورد تحلیل قرار گرفتند. یافته‌‌ها: از 22 مقاله ی مربوط  به موضوع مورد نظر: 7مقاله مربوط به میزان ثبات،6 مقاله مربوط به مصرف انرژی ،8مقاله مربوط به شاخصه های راه رفتن و 1مقاله مروری شناسایی شد که از این میان تنها 14 مورد شامل معیارهای ورود می شدند. نتیجه‌گیری: نتایج حاصل از این تحقیق نشان می دهند که تا کنون مدارک کافی جهت اثبات کارایی و برتری مفصل کنترل شونده توسط ریز پردازنده C-Leg  در مقابل سایر مکانیسم های پروتزی زانو وجود ندارد، چنان چه این ویژگی ها سابق برا ین توسط مفصل هوشمند بلچفورد Intelligent Prosthesis (IP) به اثبات رسیده بود. بنابراین شواهد و مدارک علمی بیشتری به منظور اثبات برتری C-Leg  و جایگزینی این محصول پرهزینه با سایر مفاصل سنتی زانو مورد نیاز می باشد.

Title: مروری بر همه‌گیر شناسی آسیب‌های ستون فقرات در بازیکنان گلف

http://jrrs.mui.ac.ir/index.php/jrrs/article/view/645

مقدمه : گلف ورزش محبوبی است چرا که علاوه بر نداشتن محدودیت سنی و جنسیتی، نیازمند قدرت و انعطاف بسیار زیادی نمی‌باشد. تنوع سنی و توانایی‌های متفاوت بازیکنان باعث افزایش وسعت آسیب‌های آن می‌شود. با این وجود گزارشات اندکی در خصوص آسیب گلف وجود دارد. به همین دلیل هدف اصلی این پژوهش بررسی آسیب ستون فقرات در بازیکنان گلف بود. مواد و روش‌ها: جستجوی مقالات مرتبط در سایت‌های تخصصی صورت گرفت و محدوده پوشش مقالات در زمینه‌های"Golf Injuries"، "Spine Injuries" و"Golf Spine Injuries"  از سال 1982 تا 2011 بود. یافته‌ها: یافته‌ها نشان داد میانگین بروز آسیب  تمامی نواحی بدن در رشته گلف 3/42٪ می‌باشد. شایع‌ترین محل آسیب‌دیدگی ناحیه کمر (3/18٪ تا 5/31٪) با میانگین بروز آسیب 2/26٪ می‌باشد. میزان شیوع آسیب ستون فقرات نیز 4/39٪ بوده که این عدد نشان‌دهنده شیوع نسبتا بالای آسیب ستون فقرات در این رشته ورزشی است. همچنین متوسط درصد آسیب‌های کمر در رشته گلف 33% می‌باشد. ناحیه کمر شایع‌ترین محل آسیب‌دیدگی در بین مردان و ناحیه ساعد و آرنج شایع‌ترین محل آسیب‌دیدگی در بین زنان گزارش شده است و همچنین میزان کمر درد تا حدودی در مردان بیشتر از زنان است. در مقایسه افراد حرفه‌ای و غیرحرفه‌ای ، میزان کمر درد در افراد حرفه‌ای بیشتر می‌باشد. نتیجه‌گیری: ناحیه کمر شایع‌ترین محل آسیب‌دیدگی در رشته گلف است و مکانیسم اصلی آن ترکیبی از استفاده بیش از حد و Swing اشتباه می‌باشد. جهت پیشگیری از آسیب کمر توصیه می‌شود بازیکنان مبتدی گلف به دقت به فراگیری  Swingصحیح بپردازند. همچنین مربیان می‌توانند با تمرکز بر تمرینات پایداری ناحیه مرکزی بدن در گلف بازان حرفه‌ای، احتمال وقوع آسیب ناحیه کمر را به حداقل کاهش دهند. کیلدواژه‌ها: همه‌گیرشناسی ، آسیب ستون فقرات در گلف، تاب دادن در گلف
